# Physicochemical, Antioxidant and Anticancer Characteristics of Seed Oil from Three *Chenopodium quinoa* Genotypes

**DOI:** 10.3390/molecules27082453

**Published:** 2022-04-11

**Authors:** Yingbin Shen, Liyou Zheng, Yao Peng, Xucheng Zhu, Fu Liu, Xinquan Yang, Haimei Li

**Affiliations:** 1 Innovative Center of Molecular Genetics and Evolution, School of Life Sciences, Guangzhou University, Guangzhou 510006, China; shenybin@gzhu.edu.cn (Y.S.); pengyao_79@163.com (Y.P.); zhuxc1013@gmail.com (X.Z.); 2 School of Biological and Food Engineering, Anhui Polytechnic University, Wuhu 241000, China; zhengliyou@ahpu.edu.cn; 3 Department of Food Science and Engineering, Jinan University, Guangzhou 510632, China; liu_fu@jnu.edu.cn

**Keywords:** quinoa seeds, seed oil, fatty acid, antioxidant, anticancer

## Abstract

*Chenopodium quinoa* Willd. is recognized to be an excellent nutrient with high nutritional content. However, few genotypes of quinoa were analyzed, so we found a knowledge gap in the comparison of quinoa seeds of different genotypes. This study aims to compare the physicochemical, antioxidant, and anticancer properties of seed oil from three *C. quinoa* genotypes. Seeds of three genotypes (white, red, and black) were extracted with hexane and compared in this study. The oil yields of these quinoa seeds were 5.68–6.19% which contained predominantly polyunsaturated fatty acids (82.78–85.52%). The total tocopherol content ranged from 117.29 to 156.67 mg/kg and mainly consisted of γ-tocopherol. Total phytosterols in the three oils ranged from 9.4 to 12.2 g/kg. Black quinoa seed oil had the highest phytosterols followed by red and white quinoas. The chemical profile of quinoa seed oils paralleled by their antioxidant and anticancer activities in vitro was positively correlated with the seed coat color. Black quinoa seed oil had the best antioxidant and anti-proliferation effect on HCT 116 cells by the induction of apoptosis in a dose-dependent manner, which may play more significant roles in the chemoprevention of cancer and other diseases related to oxidative stress as a source of functional foods.

## 1. Introduction

*Chenopodium quinoa* Willd. is an ancient crop locally grown in Bolivia, Peru, and Chile, but it became increasingly popular because of its characteristics such as salt tolerance and high nutrient content [1]. This crop and its products contain abundant nutritionally essential minerals, such as polyphenolics (mainly vanillic acid, ferulic acid and their derivatives as well as main flavonoids quercetin, kaempferol and their glyco sides), vitamins (ascorbic acid, thiamin, riboflavin, and α-tocopherol) and trace elements (minerals), and rich macronutrients (protein, and carbohydrate) [2,3], making it one of humanity’s most potential crops that can contribute to food security in the 21st century [4]. Meanwhile, it has been reported to possess multiple biological activities, such as anticancer activity [5], antimicrobial activity [6], and antidiabetic activity [7].

Thus, quinoa has been widely introduced in East Asia, Europe, Africa, and America with high yields, and has become a popular food resource. The seeds of this salt-resistant crop can be used to make puffed cereals or ground to flour for making bakery foods. The genus *Chenopodium* has a worldwide distribution with more than 200 species, and its seed color is variable from white to grey and black [8]. The white, red, and black quinoa (*C. quinoa*) seeds are most commonly consumed in the market, but the comparisons of chemical and biology studies are still quite limited.

An edible oil derived from quinoa seeds is a high nutritional value vegetable-based oil with some interesting properties such as antioxidant and antimicrobial effects [9]. The nutritional quality of vegetable-based oil is characterized by the fatty acid compositions and minor-components in oil [10]. It is known that the genotype can influence the chemical composition of plant seed oil [11]. On the other hand, numerous studies have reported that environmental factors as well as storage conditions show great influence on the quality characteristics of plant oil seeds [12,13].

However, there were no data published about the chemical properties, antioxidant and anticancer effects of the most consumable three genotype quinoa seeds (white, red and black, WSO, RSO, BSO). Therefore, our research aims to evaluate the nutritional and functional properties of three genotype quinoa seeds oil in different geographic areas worldwide, particularly fatty acid composition, antioxidant effects and anticancer activity. Given all these properties, this study allows for a comparison of quinoa seed oil (QSO) for their food industry.

## 2. Results

### 2.1. Physicochemical Profiles of Three QSOs

The physical and chemical properties of the three genotype extracted quinoa seed oils are presented in Table 1. The oil contents significantly differed among the tested genotypes. The oil contents of three quinoa genotype seeds ranged from 5.65 to 6.23 g/100 g, and the content of oil was highest in white quinoa seeds with an average yield of 6.19 g/100 g. The yield of quinoa seed oils by weight was 5.18–6.19% (*w*/*w*), which was averaged slightly lower than that reported previously, where it was in the range of 2% to 10% (average 5% to 7%) [14,15,16]. The seeds of the white genotype proved to be rich in oil (6.19 g/100 g on average), which is higher than red and black ones. The crude oils obtained from white, red, and black quinoa seeds were yellow, light brown, and atrovirens in appearance. All oils had R and Y Lovibond scales (Table 1). For comparison, the colors of white and red quinoa, which were expressed as R and Y Lovibond scale, displayed similar color with more yellow units (70.4 and 70 units, separately) than red (4.8 and 5 units, separately). Nevertheless, BSO display atrovirens color, which showed more yellow and less red (yellow, 73.6 units; red, 4 units). BSO showed a high ratio of Y to R values than WSO and RSO (18.4, 14.7, and 14, separately).

### 2.2. Oxidative Stability

The oxidative stability, which is defined as the resistance of oils to degrade by air, determines the quality of relative products by significantly affecting their shelf lives. The oxidation process depends on reaction conditions such as temperature, pH, catalysts, and the most important, fatty acid compositions. Oils with higher unsaturation are more susceptible to radicals.

The induction period shows the oxidative stability of edible oils, giving it the ability to reflect the oxidation state of a specific material [15]. The results in Table 1 displayed that the induction period of WSO was 1.57 h, which was significantly lower than that of RSO and BSO (7.56 and 7.50 h). According to Table 2 and the Pearson correlation analysis, a possible reason is that WSO contains less γ-tocopherol (r = 0.621), campesterol (r = 0.981), stigmasterol (r = 0.896), and β-Sitosterol (r = 0.957), which are effective antioxidants that are able to protect PUFAs in QSOs from radicals.

### 2.3. Analysis of Fatty Acids in QSOs

Table 1 shows the oil content and fatty acid composition in three genotype quinoa seeds (white, red, and black). The yield of quinoa seed oils by weight was 5.18–6.19% (*w*/*w*), and the content of oil was highest in white quinoa seeds with an average yield of 6.19 g/100 g. The predominant fatty acids in the three quinoa genotypes were PUFA (82.78–85.52%). PUFA were mainly from three essential fatty acids, linoleic acid (C18:2), oleic (C18:1), and α-linolenic acids (C18:3). Black quinoa seeds were the lowest in crude oil content but had a high level of linoleic acid (C18:2) content, giving it the highest relative PUFA content—85.52%. In addition, C24:1 and C22:6 were also found in WSO, but not detected in RSO and BSO, and C21:0 was only detected in BSO.

According to Table 2, LLO and OOL accounted for the most among the detected fatty acids in QSOss. This study provided that the primary fatty acids in QSOs were polyunsaturated fatty acids (PUFA, >80%). Based on the results from Table 1, the composition of respective fatty acids groups slightly varied among the three genotypes.

### 2.4. Analysis of Tocopherols and Phytosterols

**Figure 1:**
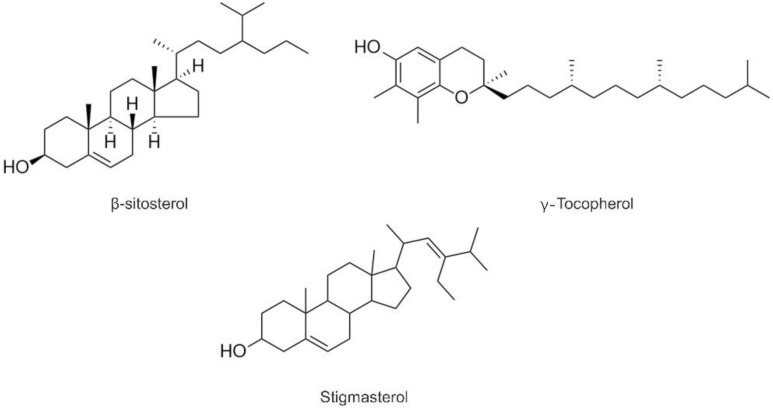
Structures of detected compounds in three QSOs.

Tocopherols are the well-known lipid soluble antioxidants and referred to as essential nutrients [16]. As shown in Table 2, four homologs (α-, β-, δ-, and γ-tocopherols) were detected in three QSOs (Figure 1), and γ-tocopherol was found to be the most abundant homologue, especially in the black ones. The total contents of tocopherols were in the range of 116.13–158.50 mg/kg, which were higher than the contents (37–60 mg/kg) reported by [14]. The black one possessed the highest content of tocopherols.

Owing to the efficacy effect on lowering blood LDL-cholesterol, phytosterols have been recognized as one of the important kinds of phytochemicals [17]. Three phytosterols (β-sitosterol, stigmasterol, and campesterol) were revealed, andβ-Sitosterol was found to be the major form, consistent with the previous results [14]. The contents of in QSQs were in the range of 8505.65–12,393.50 mg/kg.

### 2.5. FTIR Analysis of QSOs

**Figure 2:**
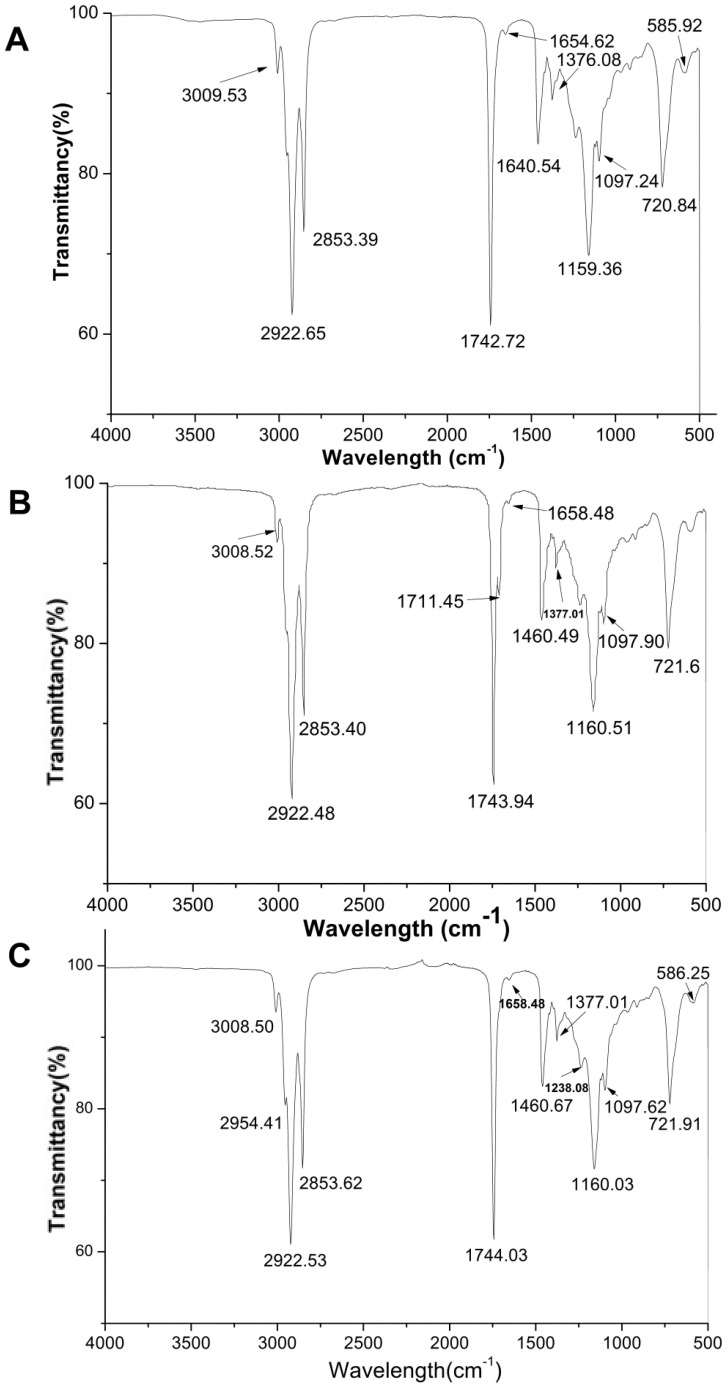
Infrared spectra of seed oils obtained from three types of *Chenopodium quinoa* Wild seed oils ((**A**) white; (**B**) red; (**C**) black).

Fourier transform infrared (FTIR) spectroscopy has been widely used to measure free fatty acids content in edible oils. A comparative study between three FTIR spectral parameters on different quinoa genotype seed oils are presented in Figure 1. In general, the major spectral peaks of three QSOs are identical and their band assignment could be determined according to the literature by Deng et al. [18] and Che Man et al. [19]. The characteristics of infrared spectra bands are summarized as follows (Figure 2): peaks at 3100–3000 cm^−1^ was ascribed to -CH stretching vibration of cis-double bonds in unsaturated fatty acids; bands at ~2922 cm^−1^ and 2853 cm^−1^ were attributed to methylene asymmetric and symmetric stretching [20]; the sharp and thin peak at 1743 cm^−1^ was an aliphatic ester -C=O stretching vibration of ester groups in triacylglycerol [21]; the band at 1658–1654 cm ^−1^ was assigned to the stretching of C=C of acyl groups of oleic and linoleic acids; a band at 1460 cm^−1^ is assigned to bending vibration of lipid CH_2_ groups; the peak at 1377 cm^−1^ was attributed to the symmetrical bending vibration of methyl groups; bands at 1160 and 1097 cm^−1^ were assigned to (C–C(=O)–O) and (OC–C) coupled bonds stretching; the major bands with the wavenumbers of ~1160 cm^−1^ was attributed to C-O stretching which indicated the hydrolysis of fatty acids and glycerols [22]; the band appeared at 1097 cm^−1^ was associated with C–O–C stretch of triglyceride ester linkage [23]; the peak at ~721 cm^−1^ was corresponding to the overlapping of the methylene rocking vibration and the out-of-plane bending vibration of cis disubstituted olefins [23].

### 2.6. Antioxidant Activities of QSOs

Table 3 shows that BSO presented the highest antioxidant activity (IC_50_: 24.6 and 40.4 mg/mL, respectively) followed by RSO (IC_50_: 74.5 and 60.2 mg/mL, respectively) and WSO (IC_50_: 102.3 and 72.1 mg/mL, respectively) by DPPH and ABTS assays. Interestingly, there was a positive correlation between the in vitro antioxidant activities and the amount of total tocopherol and main individual tocopherols of the three QSOs. Both DPPH and ABTS activities of QSOs had a significant correlation with the total phytosterols (BSO>RSO>WSO) (DPPH: r = −0.787; ABTS: r = −0.849). Similarly, high correlation coefficients between PUFA and antioxidant activity (DPPH and ABTS) were found in our study (DPPH: r = −0.998; ABTS: r = −0.985). Our results suggested that there are also PUFA in QSOs. Particularly tocopherols and phytosterols contributed most to the antioxidant activities.

### 2.7. Cell Viability and Cytotoxicity of QSOs

HCT116 cells were handled with QSOs at different concentrations for 32 h. Cell viability was measured by the MTT assay in Materials and Methods (Section 4.6). Current data in Table 3 showed that the anticancer effects of QSOs on the growth HCT116 cell line is very mild. Our results showed that the application of BSO was more efficient than WSO and RSO where IC_50_ was 281.9, 381.3, and 647.4 µg/mL, respectively. Additionally, the dose and cultivating time both influenced the inhibition of HCT116 growth. Thus, BSO may still be considered in terms of colon cancer chemoprevention.

### 2.8. BSO-Induced Morphological Changes of HCT116 Cells

**Figure 3:**
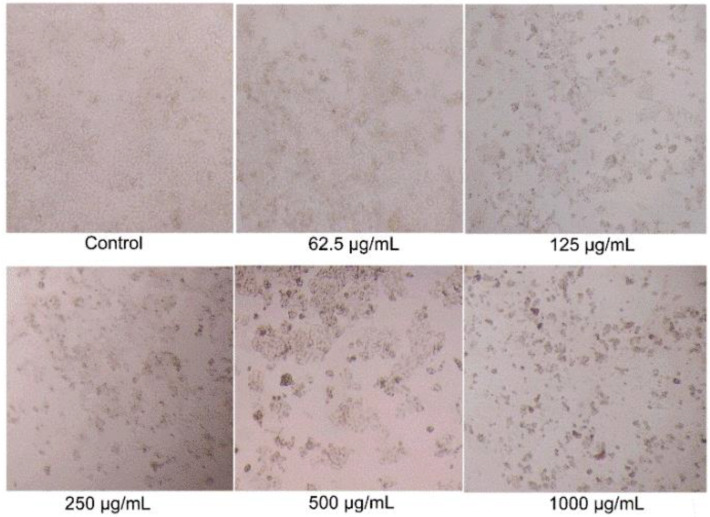
Effects of BSO (black *Chenopodium quinoa* Wild seed oil) on HCT116 cell morphology. HCT116 cells were treated with different concentrations of BSO for 36 h, and cell morphology was observed and photographed by microscopy (magnification, ×400).

While BSO showed inhibitory effects on the growth of HCT116 cells for the first time, the anticancer activity of BSO was further investigated. Firstly, the effects of BSO on the morphology of HCT116 cell was observed by the inverted microscope (Figure 3). HCT116 cells are adhesive, fusiform, and refractile cells in standard culture conditions. The architecture of untreated HCT116 cells exhibited a typical homogeneously polygonal shape (Figure 2). Figure 2 displayed that cell proliferation was significantly inhibited by BSO with numerous floating dead cells at the concentrations of 125–1000 μg/mL. The shape of individual cells changed to circular, and led to cell shrinkage after treatment with a high concentration of BSO. This is the first study to report that BSO effectively inhibits colon cancer cell growth.

### 2.9. BSO-Induced Apoptosis of HCT116 Cells

**Figure 4:**
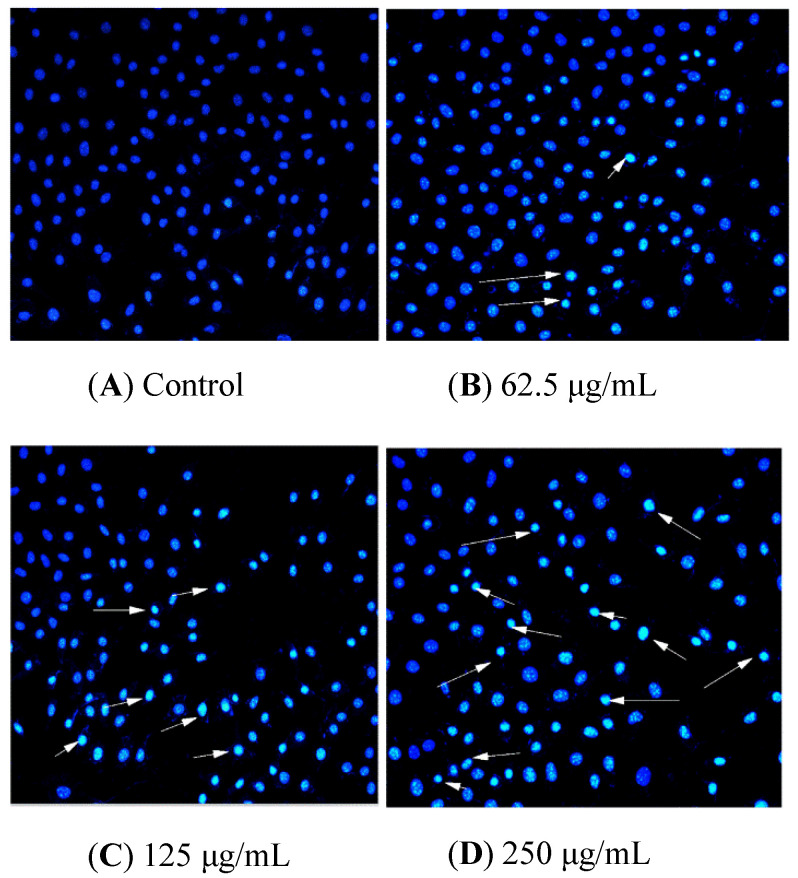
Control and BSO-treated (36 h) HCT116 cells stained with Hoechst 33258, and arrow marks indicate apoptotic cells. Hoechst 33342 staining was used to record cell apoptosis. HCT116 cells were handled with BSO at 0 (**A**), 62.5 (**B**), 125 (**C**), and 250 μg/mL (**D**) for 36 h. Apoptotic cells showed transformation in morphology in the nuclei typical apoptosis. A fluorescence microscope (200×, original magnification) was employed to photograph. Arrow-marked points are apoptotic cells.

**Figure 5:**
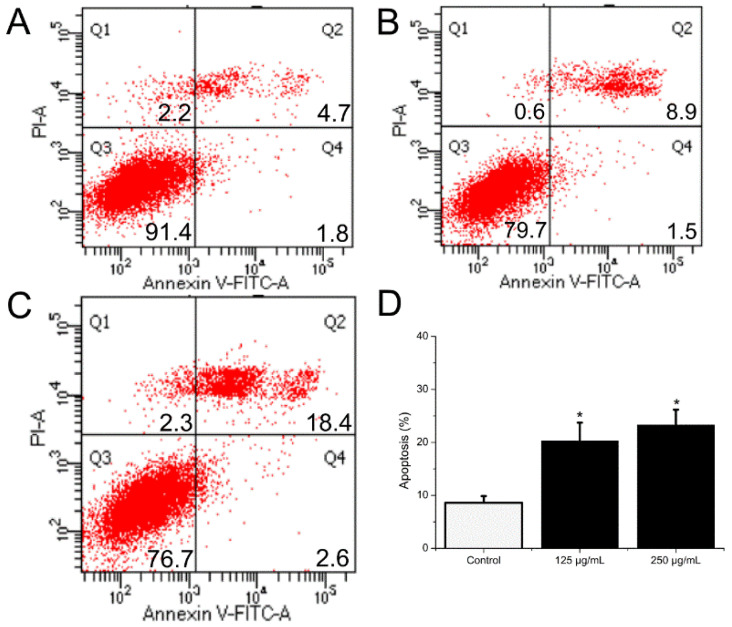
The influence of BSO on HCT116 cell apoptosis. Cells were collected after treatment and then stained with antibodies to annexin V and propidium iodide. After that, flow cytometry proceeded. BSO (125, 250 μg/mL) significantly promoted cell apoptotic. (**A**–**C**) Dot plot stained with annexin V and propidium iodide. (**D**) The percentages of apoptotic cells. As ± 3 replicate experiments were performed and the results were demonstrated in the form of means ± SD. * *p* < 0.05 versus control; * *p* < 0.05, versus negative control group. Q1, dead cells; Q2, dead/late apoptotic cells; Q3, normal cells;Q4, early apoptotic cells; Q2 + Q4, total apoptotic cells.

HCT116 cells were handled with BSO at different concentrations (0, 62.5, 125, and 250 μg/mL) for 36 h, and apoptotic status was observed by Hoechst 33342 staining. The representative images from the same sample set were shown in Figure 3. BSO induced apoptosis in HCT116 cells (white arrows) at the tested concentrations (62.5–250 μg/mL). In addition, the number of apoptotic cells increased by BSO in a dose-dependent manner. We also observed that cells were becoming round and shrunken, indicating that BSO-induced apoptosis of HCT116 cells may cause the loss of cell viability. Further quantification of BSO-induced apoptosis of HCT116 cells was conducted by flow cytometric assay. As shown in Figure 4, P2 (dead/late apoptotic HCT116 cells) +P4 (early apoptotic HCT116 cells) indicated the proportion of fully apoptotic cells. A handful of apoptotic cells was identified in the untreated group (Figure 4A) by flow cytometric assay analysis, while BSO (125 and 250 μg/mL) remarkably accelerated cell apoptosis (Figure 4C,D). The percentages of the apoptotic population in HCT116 cells treated with BSO at 125 and 250 μg/mL for 36 h were 10.4% and 21%, respectively (Figure 5). These results confirmed that BSO could induce significant apoptosis of HCT116 cells.

## 3. Discussion

The oil yield and fatty acid composition of quinoa seed oils from three quinoa genotypes were investigated in this study. The yield of quinoa seed oils by weight was 5.18–6.19% (*w*/*w*), which was averaged slightly lower than that reported previously [24,25], while similar results of fatty acid composition were obtained [25]. Usually, oils which have a low induction period are more susceptible to oxidation [26]. Seed-roasting will also affect the antioxidant ability of the oil. Our results of oxidation stability were similar to previous reports. While some of them used different measurement standards, we chose the IC_50_ value to evaluate the antioxidant capability of QSOs. However, the methods of processing, the variety of seeds, and the source of seeds had an influence on the total antioxidant capacity and oil content. [27,28,29].

Results obtained by the Rancimat analysis showed that quinoa seed oil is easily oxidized because of its unsaturation (82.8–85.5%). The main factor that limits the shelf life of seed oil is lipid oxidation, which contributes the most to its short shelf life. The induction period of white quinoa seed oil (induction period value = 1.57) was found to be lower than that of red and black genotypes (induction period value = 7.56, and 7.50 h, respectively), which indicates that red and black genotype oil have a long period of shelf life. Edible oils rich in unsaturated fatty acids are more sensitive to oxidative deterioration or autoxidation, particularly those with polyunsaturated fatty acids [30,31]. Therefore, triacylglycerol (TG), the main component of edible oil, is prone to oxidation by removing carbon–carbon double bonds. In addition, it has been shown that polyunsaturated fatty acids located at the sn-2 position are more easy to be oxidized than those in other positions [32]. The three genotypes of quinoa oils in this study contain PUFA at 82.78%–85.52%. WSO consists of 88.57% triacylglycerides, composed of the high level and diversity of sn-2 fatty acids (Table 1 and Table 2), which is the possible reason for lower induction period [33]. In particular, other antioxidant components such as free fatty acids, phospholipids, tocopherols, and phytosterols were also found in quinoa seed oils. BSO contains more tocopherols (156.67 mg/kg) and phytosterols (12245.77 mg/kg) than WSO and RSO, which may be more potent as antioxidants.

FTIR is a rapid, nondestructive, and cost-saving analytical method that reveals the basic chemical composition and physical state of total lipids [34]. In this study, FTIR absorption spectrums were applied to facilitate the presentation of differences and similarities in seed oil content and fatty acid composition of three genotype quinoa seeds. The results achieved in the spectrum presented the same type of lipid components among the three quinoa genotypes grown in different locations, which provides a comparison of the phytochemical traits of quinoa genotypes in China and Peru. Based on the results from Table 1, the composition of respective fatty acids groups slightly varied among the three genotypes. The results were also similar to other reports that analyzed the fatty acids groups of *Chenopodium quinoa* [35,36].

In our study, we found that the antioxidant capability of QSO was related to the color depth of seed coat color, and the results of three genotype quinoas were entirely different (Table 3). The current data further indicates that QSO, particularly BSO, is a potential individual antioxidants from food sources, as Marika et al. reported [37]. Solid evidence indicates that a high intake of PUFAs has been shown to decrease serum cholesterol levels and prevent cardiovascular diseases (CVDs) and CVD mortality [38,39]. A high intake of QSOs may be one of the healthiest edible oil to reduce the risk of CVDs due to the high content of PUFAs.

Up to now, there is some work reported that the extract of quinoa leaves has some anticancer activity, but no reports on that of the quinoa seed oil [40]. HCT116, the human colon cancer cell line, was employed to investigate the anticancer effects of BSO. We firstly reported that BSO has antiproliferation effects on HCT116 cells in a dose-dependent manner. Similar morphological changes, including cell shrinkage and numerous floating dead cells, were then observed in HCT116 cells when treated with BSO for 36 h. This is the first study to report that BSO effectively inhibits colon cancer cell growth.

It is a consensus that cell death could be classified into two forms: apoptosis and necrosis [41]. In human beings, apoptosis is a programmed way for cells to die [42], eliminating cancer cells while causing little damage to nearby healthy cells or tissues [43]. Therefore, induction of apoptosis in cancer cells plays an important role in cancer chemotherapeutic treatment [44]. This research showed that BSO has an anti-proliferation effect on HCT 116 cells by inducing apoptosis in a dose-dependent manner, which may be used as a functional dairy food for colon cancer prevention.

## 4. Materials and Methods

### 4.1. Reagents and Chemicals

Standards of tocopherols, phytosterols, Indeno (1,2,3-cd) pyrene (IP), and squalene were obtained from Sigma-Aldrich (St. Louis, MO, USA). Triacylglycerols were purchased from J&K Scientific (Beijing, China).

### 4.2. Extraction of C. quinoa Wild. Seed Oils

White and red quinoa seeds, cultivated in Shanxi of China, were obtained from Guangzhou Yongheng Biotechnology Company Co., Ltd. (Guangzhou, China), and the black quinoa seeds, cultivated in Peru, were purchased from Green Organic Hong Kong. The quinoa seeds (100 g) were crushed into powders and extracted with 500 mL of *n*-hexane at 60 °C for 4 h in three repetitions. The extracts were then mixed and concentrated by rotary evaporation. Quinoa seed oils from three genotypes were collected and reserved at −20 °C for the following experiments.

### 4.3. Content of Quinoa Seed Oils (QSOs)

Determination of quinoa seed oils (QSOs) content was conducted according to the standard of GB/T 14488.1-2008 [45]. The measurement unit of oil content was g/100 g seed.

### 4.4. Chemical Properties

#### 4.4.1. Oxidative Stability of QSOs

Evaluation on the oxidative stability of QSOs was conducted by the Rancimat analysis, and it was conducted with the same process as Azadmard-Damirchi’s research with the raw material switched [46].

#### 4.4.2. Color Determination

Color determination was conducted using AOCS method (Cc 13b-45) [46]. In brief, the oil samples were placed into a 25.4 mm optical path spectrophotometer cell, then tested according to the AOCS-Tintometer RY (R, red; Y, yellow) method in triplicate using the Lovibond PFX 880 Tintometer (Tintometer Group, Amesbury, UK).

#### 4.4.3. Analysis of Fatty Acid Profiles

The fatty acid composition of QSO is determined as the methyl esters of fatty acids according to Jin’s research with the raw material switched [45].

Oil samples at a concentration of 25 mg mL^−1^ (50 mg oil dissolved in 2 mL hexane) were first prepared and then esterified by adding 0.5 mL of 2 mol L^−1^ potassiumhydroxide in methanol. The fatty acid methyl esters (1.0 μL) were separated using a trace TR-FAME capillary column (0.25 μm, 60 m × 0.25 mm, Thermo Fisher, Shanghai, China) with a gas chromatograph (GC) (7820A; Agilent Technologies Inc., Santa Clara, CA, USA). Nitrogen was used as the carrier gas at a flow rate of 1 mL min. A split/splitless injector was set at a temperature of 250 °C with a split rate of 1:100, and the temperature of the flame ionization detector (FID) was 250 °C. The GC oven temperature was programed as follows: held at 60 °C for 3 min, raised to 175 °C at a rate of 5 °C min^−1^, held for 15 min, then raised to 220 °C at a rate of 2 °C min^−1^, then finally held for 10 min.

#### 4.4.4. Analysis of sn-2 Fatty Acids

The experiment on sn-2 fatty acids of QSO was performed by the same method in Shen, Y.’s research with the raw material switched [34].

#### 4.4.5. Triacylglycerol Composition

The experiment on triacylglycerol components of QSOs was performed by the AOCS method [46]. TAG composition was analyzed by using a HPLC with an evaporative light scattering detection (HPLC-ELSD) was equipped with a Lichrospher C18 column (250 × 4.6 mm, 5 µm, Hanbon, China). The relative content was reported as a percentage.

#### 4.4.6. Fourier Transform Infrared (FTIR) Spectroscopy

An FTIR spectrometer was applied to detect the 400–4000 cm^−1^ spectra of QSOs. The results were recorded as percent transmittance values.

### 4.5. Antioxidant Activity

The in vitro antioxidant effects of oils were evaluated by 1,1-diphenyl-2-picrylhydrazyl (DPPH) and 2, 2′-azino-*bis*-3-ethylbenzthiazoline-6-sulphonic acid (ABTS) assays with the same process of Shen, Y.’s research, as well as the formula which was used to calculate the scavenging effects, but the raw materials were switched [34].

### 4.6. Anticancer Activity In Vitro

The cancer cell line HCT116 (human colon carcinoma, Stem Cell Bank, Chinese Academy of Sciences, Shanghai, China) was cultivated in DMEM medium (Gibco Invitrogen, USA) containing 10% fetal bovine serum in a 5% CO_2_ incubator at 37 °C. The anticancer effects of the QSOs against HCT116 cells were detected using a 3-(4,5-dimethyl-2-yl)-2, 5 diphenyltetrazolium bromide (MTT) method. The WSO, RSO, and BSO were dissolved in DMSO and filtered through a 0.22 µm membrane (Millipore, Billerica, MA, USA). 190 µL of HCT116 (5 × 10^4^ cell/mL) was added with or without 10 µL of different concentrations of QSOs. After 36 h incubation, 20 µL MTT (5 mg/mL in PBS) was added and further incubated for 4 h. Subsequently, the supernatant was removed, and the cells and MTT crystals are dissolved in 150 µL of DMSO in each well. Lastly, the absorbance of each well measured at 570 nm using a microplate reader (Bio Tek, Broadview, IL, USA). Cell viability was calculated using the following formula: cell viability (%) = [OD_570_ (sample)/OD_570_ (control)] × 100%.

### 4.7. BSO-Induced Apoptotic Morphological Changes of HCT116 Cells

HCT116 cells were seeded in a 96-well plate and grown in conditions as above. HCT116 cells were collected 36 h after the BSO treatment at 62.5 to 1000 μg/mL, morphological changes were examined under the inverted optical microscope (Olympus, Tokyo, Japan) and photographed at 36 h.

### 4.8. Detection of Apoptotic Cells by Hoechst 33342 Staining

HCT116 cell apoptosis induced by BSO was also assayed using the Hoechst 33342 assay kit (Beyotime Institute of Biotechnology, Shanghai, China). Briefly, HCT116 cells were seeded into 6-well plates supplemented with sterile coverslips and treated with BSO (62.5, 125, and 250 μg/mL). After 36 h of culture, the medium was removed and the attached cells were carefully washed with PBS, and cells were then fixed with fresh ice-cold 4% paraformaldehyde (PFA) for 30 min at 4 °C. After fixing, cells were washed with cold PBS and incubated with Hoechst 33342 staining solution at a final concentration of 15 µg/mL for 10 min. The coverslips were subsequently washed with PBS and mounted using an anti-fade fluorescence mounting medium. Additionally, then, apoptotic cells with condensed and fragmented nuclei can be detected using fluorescence microscopy.

### 4.9. Flow Cytometric Analysis

HCT116 cells which were treated with BSO (125, and 250 μg/mL) for 36 h were collected. Then, they were fixed in ice-cold 70% (*v*/*v*) ethanol overnight at 4 °C. The cell pellet was reaped by centrifugation at 1000 rpm and resuspended in PBS. Cells were then stained with propidium iodide (PI) and analyzed using a flow cytometer (CytoFLEX, Beckman Coulter, Brea, CA, USA).

## 5. Conclusions

This study focuses on the analysis of physicochemical, phytochemical, nutritional, antioxidant, and anticancer properties of three genetically different quinoa (*C. quinoa* Willd.) genotypes of seed oils. The results obtained enriched the chemical composition and bioactivity in vitro of different quinoa genotypes (white, red, and black) seed oils and enhance the dietary use of quinoa seed oil as functional foods. Various essential lipophilic nutrients such as fatty acids, tocopherols, and phytosterols were detected in the three quinoa genotypes seeds, and the content of them increased with the quinoa seed color. The chemical profile paralleled by their in vitro bioactivities of the different quinoa cultivars suggests that quinoa seed oil, a particular dark-colored genotype, may play more significant roles in the chemoprevention of cancer and other diseases related to oxidative stress as a source of functional foods.

## Figures and Tables

**Table 1:** Comparisons of physical and chemical characteristics of seed oils of three quinoa genotypes.

	*Chenopodium quinoa* Willd. Seed		
Oil Parameters	White	Red	Black
Oil content (g/100g)	6.19 ± 0.08 ^a^	5.73 ± 0.02 ^b^	5.68 ± 0.04 ^b^
Oil stability (induction period)/h	1.57 ± 0.01 ^b^	7.56 ± 0.00 ^a^	7.50 ± 0.01 ^a^
Oil color/(units)			
Red	4.8	5.0	4.0
Yellow	70.4	70.0	73.6
Fatty acid profile (%)			
C14:0	0.42 ± 0.00 ^a^	0.21 ± 0.03 ^b^	0.15 ± 0.00 ^b^
C16:0	13.16 ± 0.14 ^a^	11.14 ± 0.02 ^b^	9.77 ± 0.04 ^c^
C16:1	1.17 ± 0.02 ^a^	0.18 ± 0.01 ^b^	-
C17:1	-	0.17 ± 0.00 ^b^	0.26 ± 0.00 ^a^
C18:0	0.85 ± 0.04 ^b^	1.25 ± 0.03 ^a^	0.69 ± 0.03 ^c^
C18:1	25.14 ± 0.07 ^a^	23.91 ± 0.07 ^b^	25.79 ± 0.01 ^a^
C18:2 Trans	0.90 ± 0.07 ^b^	1.13 ± 0.02 ^a^	0.91 ± 0.01 ^b^
C18:2	43.74 ± 0.01 ^c^	49.17 ± 0.07 ^b^	51.75 ± 0.09 ^a^
C18:3	8.24 ± 0.02 ^a^	7.09 ± 0.00 ^b^	4.59 ± 0.02 ^c^
C20:0	0.56 ± 0.02	-	-
C20:1	1.45 ± 0.03 ^b^	1.50 ± 0.00 ^b^	1.69 ± 0.00 ^a^
C20:4	0.55 ± 0.04 ^a^	0.46 ± 0.00 ^b^	0.53 ± 0.00 ^a^
C23:0	1.14 ± 0.02 ^b^	1.51 ± 0.02 ^a^	1.54 ± 0.01 ^a^
C24:0	2.67 ± 0.11 ^a^	2.28 ± 0.13 ^a^	2.34 ± 0.03 ^a^
SFA	18.87 ± 0.10 ^a^	16.4 ± 0.13 ^ab^	14.48 ± 0.09 ^b^
PUFA	52.53 ± 0.07 ^b^	56.72 ± 0.07 ^ab^	56.87 ± 0.11 ^a^
sn-2 Fatty acid composition (%)			
C16:0	1.89	2.93	2.39
C18:0	0.59	1.15	1.39
C18:1	21.69	22.2	19.97
C18:2	59.35	67.79	71.87
C18:3	6.21	5.93	3.54
C21:0	-	-	0.84
C22:6	7.89	-	-
C24:1	2.39	-	-

-: not detected. Values reported as means ± SD of three replicate analyses (*n* = 3). SFA = total saturated fatty acids, PUFA = total polyunsaturated fatty acids. ^a,b,c^: different letters in the same column indicated significant statistical differences (*p* < 0.05).

**Table 2:** Comparisons of Fat, TAGs, Tocopherols, and Phytosterols of three types of *Chenopodium quinoa* Wild seed oil.

	*Chenopodium quinoa* Wild		
	White	Red	Black
Fat compositions/%			
TAG	88.57 ± 1.51 ^ab^	81.98 ± 0.9 ^b^	91.42 ± 0.22 ^a^
1,3-DAG	0.98 ± 0.12 ^c^	4.33 ± 0.54 ^a^	2.11 ± 0.17 ^b^
1,2(2,3)-DAG	6.56 ± 0.19 ^a^	1.85 ± 0.34 ^c^	2.26 ± 0.10 ^b^
FFA	3.89 ± 1.58 ^b^	11.84 ± 0.02 ^a^	4.21 ± 0.28 ^b^
TAG compositions/%			
LLLn	3.42 ± 0.10	2.81 ± 0.03	0.87 ± 0.01
PLnLn	1.26 ± 0.04	1.04 ± 0.17	0.33 ± 0.04
MLLn	0.22 ± 0.05	-	-
PLnL	7.01 ± 0.56	9.27 ± 0.36	8.50 ± 0.24
LLL	10.78 ± 0.96	10.13 ± 0.42	6.09 ± 0.35
LnLO	1.31 ± 0.08	1.35 ± 0.03	0.41 ± 0.06
LLO	33.45 ± 0.21	31.15 ± 0.09	40.08 ± 0.13
LLP	8.46 ± 0.10	12.38 ± 0.09	10.01 ± 0.05
OOL	18.63 ± 0.14	14.48 ± 0.05	17.48 ± 0.12
PLO	9.25 ± 0.22	11.64 ± 0.01	10.78 ± 0.02
PPL	0.61 ± 0.03	1.02 ± 0.05	0.69 ± 0.04
OOP	1.09 ± 0.00	1.44 ± 0.02	1.44 ± 0.16
LOS	2.73 ± 0.03	1.63 ± 0.19	1.74 ± 0.15
OOO	1.81 ± 0.10	1.66 ± 0.06	1.59 ± 0.00
Tocopherols (mg/kg)			
α-tocopherol	38.05 ± 4.44 ^a^	33.52 ± 1.06 ^b^	34.09 ± 0.64 ^b^
β-tocopherol	2.40 ± 0.21 ^b^	1.93 ± 0.40 ^c^	3.34 ± 0.07 ^a^
γ-tocopherol	66.10 ± 1.96 ^c^	72.65 ± 0.88 ^b^	105.75 ± 0.85 ^a^
δ-tocopherol	16.53 ± 1.63	9.19 ± 1.42	13.49 ± 1.16
Total tocopherols	123.09 ± 4.32 ^ab^	117.29 ± 1.64 ^b^	156.67 ± 2.58 ^a^
Phytosterols (mg/kg)			
Campesterol	160.82 ± 8.87 ^b^	221.39 ± 21.08 ^a^	236.08 ± 1.04 ^a^
Stigmasterol	3488.37 ± 500.12 ^b^	4291.21 ± 43.64 ^ab^	4914.16 ± 177.27 ^a^
β-Sitosterol	5797.71 ± 822.15 ^b^	7664.25 ± 141.76 ^a^	7095.52 ± 32.69 ^a^
Total phytosterols	9446.91 ± 1331.14 ^b^	12176.85 ± 206.49 ^a^	12245.77 ± 208.93 ^a^

TAG, triacylglycerols; DAG, diacylglycerol; FFA, free fatty acids; P, palmitic acid; S, stearic; O, oleic; L, linoleic acid; Ln, linolenic acid. -: not detected; ^a,b,c^: various lowercase letters in the same column represented significant statistical differences (*p* < 0.05).

**Table 3:** Antioxidant activity and anticancer properties of three types of *Chenopodium quinoa* Wild seed oil.

Samples	Antioxidant Activity (IC_50_, mg/mL)		Cytotoxicity (IC_50_, μg/mL)
	DPPH	ABTS	HCT116
White	102.3 ^a^	72.1 ^a^	647.4 ^a^
Red	74.5 ^b^	63.2 ^b^	381.3 ^b^
Black	24.6 ^c^	40.4 ^c^	281.9 ^c^

^a,b,c^: Various lowercase letters in the same column represented significant statistical differences (*p* < 0.05).

## Data Availability

Not applicable.
